# Epidemiology and risk factors for mortality among methicillin-resistant *Staphylococcus aureus* bacteremic patients in Southern Brazil

**DOI:** 10.1371/journal.pone.0283774

**Published:** 2023-04-13

**Authors:** Cezar Vinícius Würdig Riche, Renato Cassol, Diego Rodrigues Falci, Mario Ramirez, Cícero Armídio Gomes Dias

**Affiliations:** 1 Departamento de Ciências Básicas da Saúde, Universidade Federal de Ciências da Saúde de Porto Alegre, Porto Alegre, Rio Grande do Sul, Brazil; 2 Serviço de Controle de Infecção, Hospital Nossa Senhora da Conceição, Porto Alegre, Rio Grande do Sul, Brazil; 3 School of Medicine, Pontifical Catholic University of Rio Grande do Sul, Porto Alegre, Rio Grande do Sul, Brazil; 4 Instituto de Microbiologia, Instituto de Medicina Molecular, Faculdade de Medicina, Universidade de Lisboa, Lisbon, Portugal; North Carolina State University, UNITED STATES

## Abstract

This study aimed to evaluate the epidemiology and 30-day mortality of adult patients with methicillin-resistant *Staphylococcus aureus* (MRSA) bacteremia. We retrospectively reviewed the demographic and clinical data of adult patients with *S*. *aureus* bloodstream infections (BSI), admitted to a tertiary public teaching medical center in Porto Alegre, Southern Brazil, from January 2014 to December 2019. A total of 928 patients with *S*. *aureus* BSI were identified in the study period (68.5 per 100,000 patient-years), and the proportion of MRSA isolates was 22% (19–27%). Thus, 199 patients were included in the analyses. The median age was 62 (IQR: 51–74) years, Charlson Comorbidity Index (CCI) median was 5 (IQR: 3–6), the Pitt bacteremia score (PBS) median was 1 (IQR: 1–4), and the most common site of infection was skin and soft tissue (26%). Most infections were hospital-acquired (54%), empirical anti-MRSA treatment was initiated in 34% of the cases, and in 44% vancomycin minimum inhibitory concentration was 1.5mg/L or above. Sixty-two (31.2%) patients died up to 30 days after the bacteremia episode. Patients with more comorbid conditions (higher CCI; aOR 1.222, p = 0.006) and a more severe presentation (higher PBS; aOR 1.726, p<0.001) were independently associated with mortality. Empiric antimicrobial therapy with an anti-MRSA regimen was associated with reduced mortality (aOR 0.319, p = 0.016). Our study identified significant risk factors for 30-day mortality in patients with MRSA BSI in a population with a high incidence of *S*. *aureus* bacteremia. Empiric treatment with an anti-MRSA drug was a protective factor. No significant variation in the incidence of *S*. *aureus* BSI was recorded throughout the period.

## Introduction

*Staphylococcus aureus* is a major human pathogen associated with a large range of infections [1]. It is one of the most common causes of bloodstream infections (BSI) worldwide, causing bacteremia with incidence ranging from 3 to 50 episodes per 100,000 person-years, with large geographical variations [2–4]. Methicillin-resistant *S*. *aureus* (MRSA) is challenging in terms of therapeutic strategies, and the occurrence of bacteremia is associated with metastatic infections, recurrence, and high mortality rates (ranging from 15% to 40%). MRSA has a global prevalence ranging from less than 5% to over 80%, being a prominent burden in many countries [5, 6]. MRSA was mostly considered a healthcare-related pathogen, although an increase in the number of community-acquired MRSA (CA-MRSA) infections is being observed in several regions. Current studies suggest that MRSA is a particularly important pathogen in developing countries [4, 7].

Several factors, such as pathogen virulence determinants, antimicrobial resistance, patients’ characteristics, and clinical management, have been studied to understand a patient’s prognosis upon MRSA bacteremia [1, 8]. Simultaneously, the morbidity of patients is increasing and resulting in more complications associated with staphylococcal infections, which may lead to poorer outcomes [9]. Unfortunately, MRSA BSI still presents high mortality and continuous improvement in care remains necessary [6, 10]. Despite the existing evidence, a better understanding of the risk factors could be important to guide therapeutic strategies. This study aimed to evaluate 30-day all-cause mortality in adult patients with MRSA BSI.

## Methods

### Study design and patient population

This was a retrospective cohort study conducted in a tertiary public teaching medical center located in Porto Alegre, Southern Brazil. The Conceição Hospital Group is a health network responsible for providing medical care to a large part of the nearly 4 million people living in the Porto Alegre metropolitan area and receiving referenced patients from the broader southern Brazil region. Adult patients (age ≥18 years old) were included if they presented MRSA bacteremia from January 2014 to December 2019. Exclusion criteria was patient data missing or inconsistent. MRSA bacteremia was defined as the presence of one or more MRSA isolates in blood cultures of patients with clinical signs of infection. Only the first episode was considered in cases of recurrent bacteremia during the study period.

### Data collection and definitions

Patient demographics and clinical data were obtained retrospectively from the Infection Control Service database and electronic medical records. The standardized data collection included: age, gender, race/ethnicity, comorbid illnesses, clinical conditions within 48 hours or at the onset of bacteremia, stay in intensive care unit, concurrent identification of other pathogens, persistence of bacteremia in consecutive blood cultures, healthcare antecedents, previous antimicrobial treatments, and empiric antimicrobial therapy. Any anti-MRSA drug with activity against the microorganism that was administered up to 24h after blood collection of the index blood culture was considered empirical anti-MRSA therapy. The sources of bacteremia were identified by reviewing the medical records, radiologic studies, and microbiological records of the patients. The Charlson comorbidity index (CCI) and Pitt bacteremia score (PBS) were calculated from patient data. Both the CCI and PBS are tools designed to predict mortality. Whereas CCI focuses on long-term mortality, PBS focuses on measuring severity of illness and predict mortality in patients with bloodstream infections by evaluating patients along 5 parameters, including: temperature, blood pressure and/or shock status, ventilator dependence, cardiac arrest, and consciousness at the onset of bacteremia [11, 12].

Infections were classified as community-acquired (CA) when the first positive culture was collected within the first 48 hours of admission and did not fulfill the criteria to be identified as a healthcare-associated (HCA) infection. A case was classified as hospital-acquired (HA) if the first blood culture was obtained 48 hours or more after hospital admission, and as HCA if the first positive culture was collected within the first 48 hours of admission but the patient was a resident of a long-term care facility, had surgery or a medical procedure in the last 90 days, or was under dialysis [13, 14]. Isolates were identified by Vitek-2 system (bioMérieux, France) and antimicrobial susceptibility test (AST) was performed according to current protocols of the Brazilian Committee on Antimicrobial Susceptibility Testing–BrCAST [15]. Vancomycin susceptibility test was performed with Etest strips (bioMérieux, France) according to the manufacturer’s instructions. These results were obtained by reviewing the microbiology laboratory records, and the antimicrobials included were cefoxitin, ciprofloxacin, clindamycin, erythromycin, fusidic acid, gentamicin, linezolid, moxifloxacin, rifampicin, tigecycline, trimethoprim-sulfamethoxazole, and vancomycin. Isolates were considered multidrug-resistant (MDR) if they were resistant to ≥3 antimicrobial classes besides β-lactams, and infections were considered polymicrobial if any other microorganism, besides skin contaminants, were also isolated. The 30-day mortality was defined as any cause of death within 30 days of the MRSA bacteremia. Additionally, the number of patients per year and patients with methicillin-susceptible *S*. *aureus* BSI admitted during the study period were counted to calculate incidences.

### Statistical analyses

Statistical analyses were conducted using the JMP software for Mac, Version 9 (SAS Institute, Cary, NC, USA). The Kolmogorov-Smirnov test was performed to check for normal distribution. Statistical differences between groups were analyzed using the chi-square or Fischer’s exact tests, for nominal and categorical data. For continuous data, Student’s t or Wilcoxon-rank-sum tests were employed. All tests were two-tailed, and a p-value of ≤0.05 was considered significant. A logistic regression model was built using a forward stepwise approach. Variables with p<0.20 were included in the model and remained in the final model if presented a p<0.05 or were deemed biologically significant. Collinearity was assessed when applicable.

### Ethics

This non-interventional study was approved by the review boards of the Conceição Hospital Group and of the Federal University of Health Sciences of Porto Alegre (CAAE: 80793917.0.0000.5530).

## Results

A total of 928 patients with *S*. *aureus* BSI were identified over the 5-year period, of which 207 were caused by MRSA. The overall incidence of BSI caused by *S*. *aureus* was 68.5 per 100,000 patient-years (range of yearly incidence: 62.2–78.3 per 100,000 patient-years), without a clear temporal trend. The proportion of MRSA isolates among *S*. *aureus* BSI was 22% (range: 19–27%), tending to decrease throughout the study period (p = 0.061). A total of 199 patients were included in the analysis of the mortality risk-factors for MRSA BSI.

### Clinical and demographic data

The patients were predominantly male (64.3%), the median age was 62 years with an interquartile range (IRQ) of 51 to 74 years, and white (78.9%). The median of CCI was 5 (IQR: 3–6), the most frequent comorbidity was hypertension (45.2%), followed by diabetes mellitus (31.6%) and cardiomyopathy (coronary artery disease or congestive heart failure; 21.1%). The median PBS was 1 (IQR: 1–4). Most infections were HA (54.3%), of which 25.0% were in the ICU when diagnosed, while 27.6% of cases were CA. The most frequent primary site of infection among CA-MRSA cases was the skin and soft tissues, being responsible for 28 cases (50.9%), followed by pulmonary infections (12 cases; 21.8%). The most frequent site of infection in HCA and HA cases was catheter-related, with 41 cases (28.5%). See Table 1 for further details.

**Table 1:** Univariate and multivariate analysis of risk factors for 30-day all-cause mortality in patients with MRSA bacteremia.

		30-day outcome				
Variable	TOTAL, No. (%)	Survivors 137 (68.8)	Deaths, 62 (31.2)	p value	30-day mortality aOR (95% CI)	p value
Age, in years ^a^ (IQR)	62 (51–74)	61 (51–71)	67 (55–77)	**0.020**		
Male gender	128 (64.3)	86 (62.7)	42 (67.7)	0.498		
Race/ethnicity						
White	157 (78.9)	107 (78.1)	50 (80.6)	0.135		
Black	29 (14.6)	18 (13.1)	11 (17.7)			
Other	13 (6.5)	12 (8.7)	1 (1.6)			
Comorbid conditions						
Hypertension	90 (45.2)	62 (45.2)	28 (45.1)	0.990		
Diabetes mellitus	63 (31.6)	46 (33.6)	17 (27.4)	0.387		
Cerebrovascular	21 (10.5)	16 (11.7)	5 (8.0)	0.442		
Dementia	25 (12.5)	15 (10.9)	10 (16.1)	0.307		
CAD	28 (14.7)	20 (10.0)	8 (12.9)	0.750		
Heart failure	23 (11.5)	14 (10.2)	9 (14.5)	0.379		
Asthma or COPD	23 (11.5)	16 (11.7)	7 (11.3)	0.936		
CRD	30 (15.0)	22 (16.0)	8 (12.9)	0.564		
Liver disease	9 (4.5)	3 (2.2)	6 (9.7)	0.275		
Malignancy	36 (18.1)	21 (15.3)	15 (24.2)	**0.132**		
HIV or AIDS	22 (11.0)	13 (9.5)	9 (14.5)	0.294		
Alcohol abuse	32 (16.1)	25 (18.2)	7 (11.3)	0.215		
Tobacco	66 (33.1)	54 (39.4)	12 (19.3)	**0.005**		
Epidemiology						
CA	55 (27.6)	34 (24.8)	21 (33.9)	0.422		
HCA	36 (18.1)	26 (19.0)	10 (16.1)			
HA	108 (54.3)	77 (56.2)	31 (50.0)			
CCI ^a^ (IQR)	5 (3–6)	4 (2–6)	5 (4–7)	**0.004**	1.222 (1.061–1.409)	0.006
ICU	36 (18.0)	22 (16.0)	14 (22.6)	0.268		
PBS ^a^ (IQR)	1 (1–4)	1 (1–3)	4 (3–5)	**<0.001**	1.726 (1.410–2.112)	<0.001
ATM 30 days	98 (49.2)	65 (47.4)	33 (53.2)	0.450		
Previous treatment with vancomycin	26 (13.2)	23 (16.9)	3 (4.9)	0.215		
Polymicrobial	16 (8.0)	7 (5.1)	9 (14.5)	**0.004**		
Persistent bacteremia	43 (21.6)	32 (23.3)	11 (17.7)	0.372		
Empiric MRSA treatment	67 (33.8)	54 (39.7)	13 (21.0)	**0.009**	0.319 (0.126–0.806)	0.016
Infection site						
SST	44 (26.1)	34 (27.8)	10 (21.7)	**0.007**		
Catheter	41 (24.4)	36 (29.5)	5 (10.8)			
Pulmonary	39 (23.2)	23 (18.8)	16 (34.7)			
Surgical site	12 (7.1)	10 (8.2)	2 (4.3)			
Blood non-CRI	9 (5.4)	7 (5.7)	2 (4.3)			
Abdominal	9 (5.4)	3 (2.4)	6 (13.0)			
Urinary	9 (5.4)	7 (5.7)	2 (4.3)			
Not identified	6 (3.0)	2 (1.6)	3 (6.5)			
MDR isolate	70 (35.1)	50 (36.5)	20 (32.2)	0.674		
Vancomycin MIC ≥1.5mg/L	87 (43.7)	65 (47.4)	22 (35.4)	**0.155**		

Abbreviations: CA: community-acquired; HCA: healthcare-associated; IQR: interquartile range; CCI: Charlson comorbidity index; CAD: coronary artery disease; COPD: chronic obstructive pulmonary disease; CRD: chronic renal disease; HA: hospital-acquired; ICU: intensive care unit; PBS: Pitt bacteremia score; ATM: antimicrobial; MRSA: methicillin-resistant S. aureus; SST: skin and soft tissue; CRI: catheter-related infection; MDR: multidrug resistant microorganism; MIC: minimum inhibitory concentration.

^a^ Data presented in median and IQR.

A total of 49.2% of the patients had received treatment with an antimicrobial in the previous 30 days, and 13.2% had received at least one day of treatment with vancomycin. Empirical antimicrobial therapy with activity against MRSA was administered to 67 (33.8%) patients. A total of 21.6% of the patients (43 individuals) presented persistent bacteremia, and 8.0% had polymicrobial infections. The 30-day mortality was 31.2%, and the in-hospital mortality (all-cause) was 39.6%.

### Antimicrobial susceptibility

Only 3 isolates did not present resistance to antibiotics other than β-lactams. Resistance was observed against erythromycin (72.3%), ciprofloxacin (49.7%), clindamycin (43.7%), moxifloxacin (37.6%), gentamicin (22.1%), trimethoprim-sulfamethoxazole (11.0%), rifampicin (10.5%), linezolid (1.0%), and fusidic acid (0.5%). All isolates were susceptible to tigecycline. The minimum inhibitory concentration (MIC) of vancomycin ranged from 0.25 to 2.0mg/L, and the proportion of isolates with MIC ≥1.5mg/L was 43.7%. A total of 70 (35.1%) isolates were classified as MDR and the proportion of MDR was 10.9% among CA-MRSA cases, and 36.1% and 47.2% among HCA and HA-infections, respectively.

### Mortality associated risk-factors

Results from univariate and multivariate logistic regression models evaluating the association between risk factors and 30-day mortality are shown in the table. The CCI (aOR 1.222, p = 0.006) and PBS (aOR 1.726, p<0.001) were independent risk factors associated with increased 30-day mortality. Our univariate analyses indicated that other factors were associated with increased mortality, such as age, presence of malignancy, tobacco use, presence of polymicrobial infection, primary site of infection, and vancomycin MIC. However, these associations were not significant in multivariate analyses. The use of empirical antimicrobial therapy with activity against MRSA was independently associated with reduced 30-day mortality (aOR 0.319, p = 0.016).

## Discussion

Only a few studies have evaluated the burden of MRSA infections in Latin America, and most countries do not have a continuous surveillance program. Our study revealed higher incidence than those observed in developed countries, and the overall incidence of 25 per 100,000 persons found in the SENTRY Surveillance Program [3], highlighting the importance of *S*. *aureus* as a cause of bloodstream infection in this population. Despite the high incidence of *S*. *aureus*, the prevalence of MRSA isolates was lower than that reported in other studies. The World Health Organization global antimicrobial resistance report presented a proportion of 43% to 45% MRSA among invasive *S*. *aureus* isolates in the Americas [WHO, 2014] [5]. A similar data was described in a Latin American observational study, which reported an overall proportion of 44.7% [7]. In 2011 the Brazilian SCOPE, a multicenter study of nosocomial BSI, found that *S*. *aureus* was the leading microorganism in the country, being responsible for 15.4% of all infections [16]. The same study reported a proportion of 43.7% MRSA and a crude mortality of 31.0% associated with *S*. *aureus* BSI. These may reflect the differences in MRSA epidemiology according to regions, potentially due to differences in the circulating MRSA clones [17]. Despite its lower proportion among *S*. *aureus* bacteremias in Porto Alegre, MRSA remains an important pathogen and continuous surveillance is needed to monitor if the reduction trend is confirmed.

Our study found a high prevalence of CA-MRSA infections, most of which were related to skin infections. CA-MRSA have been described as causing severe infections, usually pneumonia and skin and soft tissue infections in individuals not exposed to healthcare settings [18]. While in some regions of the world MRSA remains mostly an HA and HCA pathogen, in others it has become endemic in the community, and in Latin America, it is an increasing problem [19]. A large national study of *S*. *aureus* infections in China showed a 24% proportion of CA-MRSA, with regional differences, ranging from 5 to 40% [20]. In 2018, a Latin American observational study reported an occurrence of only 8.6% of CA-MRSA infections, with a low prevalence in Brazil [7]. A systematic review with data from Brazil, Argentina, Uruguay, Guyana, French Guiana, and Mexico showed CA-MRSA infection ranging from 0 to 87.9%, where Argentina presented the highest prevalence, followed by Mexico (51.1%) [21]. In this same systematic review, the CA-MRSA prevalence in Brazil ranged from 4.5 to 8.6%, reaching 25% in a study of skin infections in patients with dermatological conditions [21]. Our data showed a much higher prevalence of CA-MRSA, a fact that should be taken into account when deciding on empirical antimicrobial therapy in our setting.

We found a significant proportion of MDR MRSA isolates, but mostly associated with HCA and HA infections, which further limits treatment options. Despite this, linezolid, vancomycin and tigecycline remain good treatment options. A similar proportion of MDR was reported previously in a study from southern Brazil with 42.9% of MDR among MRSA isolates [22]. This is a deepening problem, with infections caused by MDR MRSA increasing in several countries [23]. We found a high proportion of isolates with vancomycin MIC ≥1.5mg/L, in line with the MIC creep phenomenon we had described previously [24]. Genomic studies are necessary for a better understanding of the clones responsible and for the genetic determinants of these increased vancomycin MIC values over time.

Our data showed that elevated CCI and PBS were independently associated with 30-day mortality, findings consistent with the literature. The presence and severity of underlying diseases are also responsible for increased mortality following *S*. *aureus* bacteremia [25]. In the Seas and colleagues cohort study, CCI was reported as an independent factor associated with all-cause mortality in patients with *S*. *aureus* BSI [7]. More so, previous studies have also found PBS as a predictor of mortality in *S*. *aureus* BSI. In a retrospective study of patients with bacteremia caused by MRSA with reduced vancomycin susceptibility (MIC = 2.0mg/L), CCI and PBS were reported as independent factors associated with increased 30-day mortality [26]. PBS was also an independent mortality predictor in two prospective cohort studies [7, 27]. Taken together these suggest that the initial clinical presentation of *S*. *aureus* BSI should be considered to evaluate the potential severity of the infection. We failed to find factors related to the bacteria (MDR or vancomycin MIC) as associated with 30-day mortality in this cohort. In other words, vancomycin MIC ≥1.5mg/L lost significance once clinical covariates were adequately controlled. These findings reinforce the notion that MRSA infection mortality is more influenced by the severity of the illness and patient comorbidities than by characteristics of the pathogen itself, although we cannot exclude that bacterial characteristics for which we did not test could impact mortality [9, 28].

In our study, empiric treatment with an anti-MRSA drug was an independent protective factor. It is well known that an early identification and prompt administration of the appropriate antimicrobials improve patient outcomes in sepsis. *S*. *aureus* bacteremia is one of the most serious bacterial conditions and delay in therapy is associated with treatment failure and the occurrence of complications [29, 30]. Many patients in our study did not present traditional risk factors to suspect an MRSA infection [1]. We propose that the empiric use of an anti-MRSA therapy should be considered for patients with more comorbid conditions and patients with higher PBS, due to their increased mortality and the possibility of an MRSA infection. We did not evaluate the specific drug used in empiric therapy targeting MRSA; however, in this center, vancomycin and linezolid are key tools to treat MRSA.

This study has limitations inherent to its retrospective design. Also, our data represents one public health network, which, although responsible for a large population, may not be representative of the current trends and burden in the whole Southern Brazil. The strengths of this study include the large period evaluated and the diversity of infections studied, including community-acquired infections.

In this retrospective cohort study, we described the clinical characteristics of MRSA BSI over a large period and its impact on 30-day mortality. The Charlson comorbidity index and the Pitt bacteremia score were significant predictors of 30-day mortality in these patients, and empiric treatment with an anti-MRSA drug was found to be protective. Patients with high PBS or with more comorbid conditions, in settings with a moderate frequency of MRSA, should receive prompt management for infection, possibly including antimicrobial treatment with an anti-MRSA agent. This may be even more relevant in settings with a high burden of CA-MRSA infections, such as ours. Given the heterogeneity in epidemiology and the dynamic nature of MRSA infections in Brazil, sustained and larger epidemiological surveys are needed to guide clinical management of these important infections.

## Supporting information

S1 Data (XLSX)
